# Monocyte populations as markers of response to adalimumab plus MTX in rheumatoid arthritis

**DOI:** 10.1186/ar3928

**Published:** 2012-07-27

**Authors:** Luis Chara, Ana Sánchez-Atrio, Ana Pérez, Eduardo Cuende, Fernando Albarrán, Ana Turrión, Julio Chevarria, Miguel A Sánchez, Jorge Monserrat, Antonio de la Hera, Alfredo Prieto, Ignacio Sanz, David Diaz, Melchor Alvarez-Mon

**Affiliations:** 1 Department of Medicine, University of Alcalá, N-II km 33, Alcala de Henares 28871, Spain; 2 Immune System Diseases and Oncology Service, University Hospital "Príncipe de Asturias", N-II km 33, Alcala de Henares 28871, Spain; 3 Division of Allergy, Immunology and Rheumatology, Department of Medicine, University of Rochester, 601 Elmwood Ave, Rochester, NY 14642, USA

## Abstract

**Introduction:**

The treatment of rheumatoid arthritis (RA) patients with anti-tumor necrosis factor alpha (TNFα) biological drugs has dramatically improved the prognosis of these patients. However, a third of the treated patients do not respond to this therapy. Thus, the search for biomarkers of clinical response to these agents is currently highly active. Our aim is to analyze the number and distribution of circulating monocytes, and of their CD14^+high^CD16^-^, CD14^+high^CD16^+ ^and CD14^+low^CD16^+ ^subsets in methotrexate (MTX) non-responder patients with RA, and to determine their value in predicting the clinical response to adalimumab plus MTX treatment.

**Methods:**

This prospective work investigated the number of circulating monocytes, and of their CD14^+high^CD16^-^, CD14^+high^CD16^+ ^and CD14^+low^CD16^+ ^subsets, in 35 MTX non-responder patients with RA before and after three and six months of anti-TNFα treatment using multiparametric flow cytometry. The number of circulating monocytes in an age- and sex-matched healthy population was monitored as a control.

**Results:**

Non-responder patients with RA show an increased number of monocytes and of their CD14^+high^CD16^-^, CD14^+high^CD16^+ ^and CD14^+low^CD16^+ ^subsets after three months of adalimumab plus MTX treatment that remained significantly increased at six months. In contrast, significant normalization of the numbers of circulating monocytes was found in responders at three months of adalimumab plus MTX treatment that lasts up to six months. CX3CR1 expression is increased in monocytes in non-responders. At three months of anti-TNFα treatment the number of circulating monocytes and their subsets was associated with at least 80% sensitivity, 84% specificity and an 86% positive predictive value (PPV) in terms of discriminating between eventual early responders and non-responders.

**Conclusions:**

The absolute number of circulating monocytes and of their CD14^+high^CD16^-^, CD14^+high^CD16^+ ^and CD14^+low^CD16^+ ^subsets at three months of adalimumab plus MTX treatment, have a predictive value (with high specificity and sensitivity) in terms of the clinical response after six months of anti-TNFα treatment in patients with RA.

## Introduction

Dramatic improvements in the management of patients with rheumatoid arthritis (RA) have been achieved in the last two decades. The possibilities of controlling disease progression and joint destruction have greatly increased through the use of biological drugs with tumor necrosis factor alpha (TNFα) blockade activity [1,2]. In addition, new biologic therapies with different targets, such as interleukin (IL)-6, CD20, have shown relevant effectiveness in the control of RA [3,4]. This expansion in the number of effective therapies is also accompanied by a growing evidence of wide variation in the RA patient clinical response to these biological therapies [5]. The prevention of delays in the use of the most effective treatment for each patient, the avoidance of unnecessary secondary effects and the rational use of scant economic resources have all stimulated the search for biomarkers that predict the response of individuals to different RA treatments.

Monocytes are bone marrow-derived cells that mediate essential regulatory and effector functions in innate and adaptative immunity [6]. Circulating peripheral blood monocytes may migrate into tissues where they differentiate into different effector cells, such as macrophages, dendritic cells and osteoclasts [6-9]. The circulating monocyte compartment is phenotypically and functionally heterogeneous. Three major subsets based on the expression of CD14 (the lipopolysaccharides (LPS) co-receptor) and CD16 (the FcγRIII low affinity immunoglobulin G (IgG) receptor) have been defined in circulating monocytes [6,8-10]. The majoritarian subsets or "classic" monocytes are phenotypically defined by an intense expression of CD14, but lack CD16 (CD14^+high^CD16^-^). The minoritarian subsets (10% of the circulating monocytes) are characterized by the expression of CD16 plus either high or low levels of CD14 (intermediate CD14^+high^CD16^+ ^monocytes and CD14^+low^CD16^+ ^non-classical monocytes, respectively) [11]. These three phenotypically defined monocyte subsets show different functional properties, such as patterns of cytokine secretion and chemokine receptor expression, and migratory properties into normal and inflamed tissue. Furthermore, these three different monocyte subsets also differ in their ability to differentiate into effector cells, including macrophages, dendritic cells and osteoclasts [8-10]. Monocytes and monocyte derived cells appear to be involved in the pathogenesis of RA [12,13].

Roughly, 20 to 30% of RA patients show unresponsiveness to anti-TNFα biological therapy [14,15]. These therapeutic failures may occur early after the start of treatment or late in a secondary phase that develops in initial responders during the course of therapy [16]. The latter appears to be related to the formation of anti-drug antibodies (anti-anti-TNFα antibodies) in a subset of patients [17]. However, the mechanism of early anti-TNFα treatment resistance remains elusive. Thus, the hypothesis tested in this work was that the pre-treatment absolute number, distribution and migratory properties of circulating monocytes, and of their CD14^+high^CD16^-^, CD14^+high^CD16^+ ^and CD14^+low^CD16^+ ^subsets, might help predict the early therapeutic response to anti-TNFα biological therapy. The number of CD14^+high^CD16^-^, CD14^+high^CD16^+ ^and CD14^+low^CD16^+ ^monocytes was prospectively investigated in patients with RA before initiation of anti-TNFα therapy and during the first six months of anti-TNFα treatment. Furthermore, and with respect to active naive RA patients and healthy donors, we investigated the pattern of circulating monocyte subsets in patients with active RA before starting treatment with adalimumab plus MTX. This allowed the potential link between the activity of the disease and the pattern of abnormality in this cellular compartment to be studied.

## Materials and methods

### Inclusion and exclusion criteria

Thirty-five patients visiting the Immunology and Rheumatology Service at the Hospital Universitario Príncipe de Asturias (HUPA) were enrolled in the study. All gave their informed consent to be included; the study was approved by the hospital's clinical ethics committee. Patients were studied in parallel with sex- and age-matched healthy controls.

#### Inclusion criteria

The entry criteria included patients who had: 1) a diagnosis of RA according to the 1987 revised European League Against Rheumatism (EULAR) criteria with an of age higher than 18 years [18]; 2) a disease activity score 28 (DAS28) according to EULAR criteria of more than 2.5 [18]; and 3) to be treated with weekly MTX (15 to 20 mg per week) for at least the previous three months.

#### Exclusion criteria

The exclusion criteria for this study included severe cardiovascular disease (congestive heart failure, uncontrolled hypertension, coronary disease, severe arrhythmia), hematopoietic, lung, hepatic or renal disorders not related to the RA, diabetes mellitus, active bacterial or viral infections, other autoimmune diseases, treatment with glucocorticoids, immunosuppressors or other drugs that interact with the immune system in the previous three months, treatment with steroids in the previous month, possible pregnancy or lactation during the six-month study period, simultaneous malignancy, malnutrition and congenital immunodeficiency.

We also included in the study 13 patients who were age ≥18 years, had a diagnosis of RA according to the 1987 revised European League Against Rheumatism (EULAR) criteria [18], had a Disease Activity Score 28 (DAS28) according to EULAR criteria of more than 2.5 [18], and were untreated with disease-modifying antirheumatic drugs (DMARDS).

### Study protocol

All patients were treated weekly for at least three months with 15 to 20 mg MTX (orally) plus 20 mg folic acid the next day and adalimumab 40 mg every other week. Patients were also advised to take non-steroidal anti-inflammatory drugs at fixed doses during the study. All were monitored monthly for clinical and analytical tolerance to MTX and adalimumab treatment and at three and six months to assess clinical response and to undertake immunological studies. Disease activity was determined by the DAS28 score according to EULAR criteria and using a validated Spanish version of the Health Assessment Questionnaire (HAQ) [19]. The clinical response of the patients to MTX plus adalimumab treatment was defined according to EULAR criteria for RA [18], classifying patients as responders or non-responders. The responder group included those patients with a DAS28 score of < 2.6 after six months of MTX plus adalimumab treatment, plus those whose DAS28 score decreased by at least 1.2 with respect to the initial value.

Three peripheral blood samples were taken from each patient by antecubital venipuncture at baseline (before starting adalimumab treatment), at three months of treatment and at six months of treatment.

### Isolation of peripheral blood mononuclear cells

Peripheral blood mononuclear cells (PBMC) were separated out by Ficoll-Hypaque (Lymphoprep™, Axis-Shield, Oslo, Norway) gradient centrifugation [20]. They were then resuspended in Roswell Park Memorial Institute medium (RPMI) 1640 (Biowhittaker Products, Verviers, Belgium) supplemented with 10% heat-inactivated fetal calf serum, 25 mM Hepes (Biowhittaker Products) and 1% penicillin-streptomycin (Biowhittaker Products). Cell enumeration was performed by conventional light microscopy using a Neubauer chamber following trypan blue dead cell exclusion criteria. The viability of fresh PBMC was checked by both trypan blue (light microscopy) and 7-aminoactinomycin D (7-AAD) (flow cytometry) exclusion.

### Immunophenotype studies

For immunofluorescent staining, fresh monocytes were incubated with a combination of fluorescein (FITC), phycoerythrin (PE), peridinin chlorophyll protein conjugate (PerCP), and Alexa Fluor-647-labeled monoclonal antibodies (MoAbs). The MoAbs were used in a four-color combination (FITC/PE/PerCP/Alexa Fluor-647): CX3CR1/-/CD14/CD16. Control studies with unstained cells and cells incubated with isotype-matched irrelevant FITC-, PE-, PerCP and Alexa Fluor-647-labeled MoAbs were performed for each experiment. For these procedures, anti-CD14 and anti-CD16 were purchased from Becton Dickinson (Franklin Lakes, NJ, USA) and anti-CX3CR1 purchased from MBL, Medical and Biological Laboratories Co., Ltd (Naka-ku Nagoya, Japan). Cell acquisition and four-color immunofluorescence analyses were performed using a FACSCalibur flow cytometer (Becton Dickinson) running CellQuest Pro (Becton Dickinson) and FlowJo software (Tree Star, Inc., Ashland, OR, USA), respectively. In the FSC-SSC dot plot, a biparametric gate was drawn around the monocyte population. The absolute number of circulating monocyte subsets was calculated by the percentage of each subpopulation in peripheral blood determined by flow cytometry multiplied by the total number of monocytes per microliter measured by Beckman Coulter, Inc. (Brea, CA, USA).

### Statistical analysis

Variables with nominal scale are described using absolute and relative frequencies. The Kolmogorov-Smirnov test was employed to verify the normality of distribution of continuous variables. For univariate description of normally distributed clinical variables, mean values and standard deviation (SD) are given. The differences in demographic characteristics were assessed using Pearson's chi-squared tests, Fisher's exact test, Student's t-tests or ANOVA with Bonferroni post-hoc adjustment for multiple testing. The results of the immunophenotype studies were expressed as mean and the standard error of the mean (SEM). Comparisons among healthy controls, responders and non-responders at baseline, were carried out using the Kruskal-Wallis test or ANOVA test for different samples. We used a two-sided analysis of variance (ANOVA) with Bonferroni adjustment to evaluate longitudinal changes. To assess the value of baseline circulating monocytes and their different subsets as predictors of MTX treatment response at six months of follow-up, receiver operating characteristic (ROC) curve analyses were performed, and the respective areas under the curves (AUC) were determined as measures of overall performance. The best predictive cut-off value was defined as that which gave the highest product of sensitivity and specificity, positive predictive value (PPV) and negative predictive value (NPV). All analyses were performed using the Statistical Package for the Social Sciences (SPSS, version 19.0, Chicago, IL, USA). Significance was set at P < 0.05.

## Results

### Demographic characteristics of the patients

Table 1 shows the baseline characteristics of the 23 responders and 12 non-responders included in the analysis. No significant differences were seen between these groups of patients with respect to age or sex distribution, nor in the clinical or analytical variables studied.

**Table 1:** Demographic, clinical and biological data of the patients at baseline

Variables	MTX active (*n *= 13) (mean ± SD)	Responders (*n *= 23) (mean ± SD)	Non-responders (*n *= 12) (mean ± SD)	*P*-value
Age (years)	51.64 ± 10.88	50.36 ± 5.82	52.25 ± 11.09	.762
Sex (men/women)	38.46%/61.54%	33.33%/66.67%	37.50%/62.50%	.809
CRP (mg/dl)	16.61 ± 7.21	14.33 ± 14.10	11.68 ± 10.74	.569
Rheumatoid factor (+/-)	92.30%/7.70	80.00%/20.00%	62.50%/37.50	.283
Anti-CCP (UI/ml)	423.25 ± 296.01	994.60 ± 620.24	329.50 ± 7.50	.075
DAS28	3.71 ± .71	2.64 ± .84	3.20 ± .88	.090
Erosions (+/-)	30.76%/69.24%	60.00%/40.00%	71.40%/28.60%	.518
Onset of Symptoms (months)	9.60 ± 9.10	100.53 ± 66.14	148.14 ± 77.79	.090
HAQ	.78 ± .53	1.18 ± .47	1.31 ± .72	.553

Anti-CCP, anti-cyclic citrullinated peptide antibody; CRP, C-reactive protein; DAS28, Disease Activity Score 28; HAQ, Health Assessment Questionnaire

### Non-responder RA patients show increased number of monocytes and of their CD14^+high^CD16^-^, CD14^+high^CD16^+ ^and CD14^+low^CD16^+ ^subsets after three months of anti-TNFα treatment

Next, we investigated the absolute number of circulating monocytes, and those of the CD14^+high^CD16^-^, CD14^+high^CD16^+ ^and CD14^+low^CD16^+ ^subsets were studied in all 35 MTX-treated patients before starting adalimumab plus MTX treatment, and again at three and six months.

**Figure 1:**
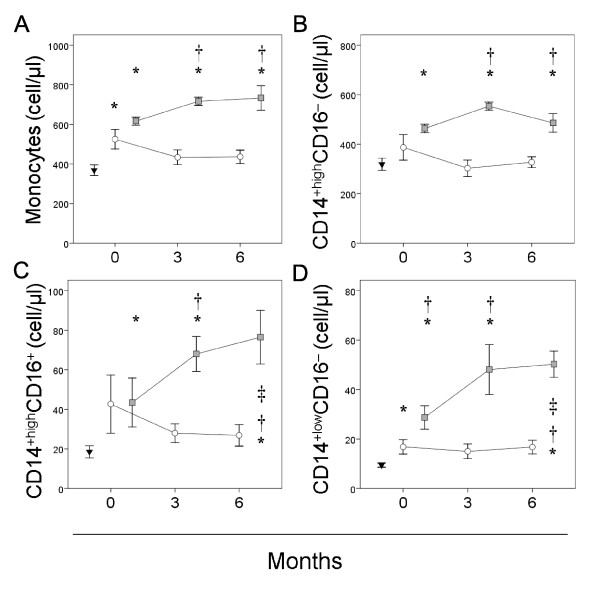
**Percentage of circulating monocytes in patients with RA at baseline and over anti-TNFα treatment**. The absolute number (cells/μl) of circulating monocytes (panel A), CD14^+high^CD16^- ^(panel B), CD14^+high^CD16^+ ^(panel C) and CD14^+low^CD16^+ ^monocytes (panel D) of non-responders (□) and responders (○) at baseline and after three and six months of MTX treatment, and of healthy controls (▼), are shown as the mean ± SEM. * significant difference between patients with RA and healthy controls. † significant difference between non-responders and responders. ‡ significant difference between baseline and six-month values.

Figure 1 shows that the absolute number of circulating monocytes in the non-responders was significantly higher than in the responders at three months of adalimumab plus MTX treatment. The absolute number of monocytes in the non-responders remained significantly increased with respect to responders and healthy controls until the six-month study period. In contrast, significant normalization of the numbers of circulating monocytes was found in responders at three months of anti-TNFα treatment that lasts up to six months. We did not find significant differences between responders and non-responders at baseline but both were significantly higher with respect to healthy donors.

The non-responders also had significantly higher numbers of circulating monocytes and their CD14^+^CD16^-^, CD14^+high^CD16^+ ^and CD14^+low^CD16^+ ^subsets than healthy controls at baseline and all over the six months of anti-TNFα treatment. However, significant differences between responders and non-responders appeared at three months and remained until the end of the study of adalimumab plus MTX treatment for CD14^+^CD16^- ^and CD14^+high^CD16^+ ^subsets. In contrast, the number of circulating CD14^+low^CD16^+^monocytes was significantly increased in non-responder patients with respect to healthy donors and responders at baseline and along the six months of study. No significant differences were found between responders and healthy controls in the absolute number of CD14^+^CD16^-^, CD14^+high^CD16^+ ^and CD14^+low^CD16^+ ^subsets at any time of the study but the CD14^+low^CD16^+ ^subset was significantly increased in responder patients at baseline.

### Non-responders show progressive redistribution of the monocyte subsets

**Figure 2:**
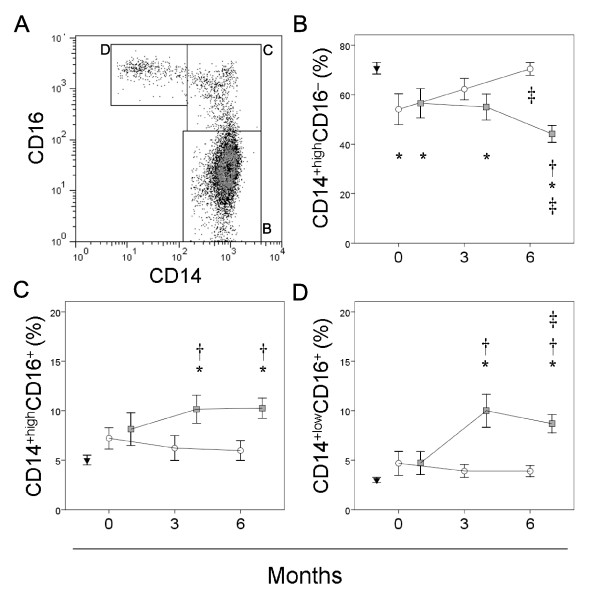
**Percentage of monocyte subsets in patients with RA at baseline and over anti-TNFα treatment**. Panel A represents flow cytometry analysis of circulating monocytes from a representative patient. The percentage of circulating CD14^+high^CD16^- ^(panel B), CD14^+high^CD16^+ ^(panel C) and CD14^+low^CD16^+ ^monocytes (panel D) of non-responder (□) and responders (○) at baseline and after three and six months of MTX treatment, and those of healthy controls (▼), are shown as mean ± SEM. * significant difference between patients with RA and healthy controls. † significant difference between non-responders and responders. ‡ significant difference between baseline and six-month values.

Figure 2 shows the distribution of the CD14^+high^CD16^-^, CD14^+high^CD16^+ ^and CD14^+low^CD16^+ ^monocyte subsets in the patients and healthy controls. Responders and non-responders showed a significantly smaller percentage of CD14^+high^CD16^- ^monocytes at baseline with respect to healthy donors but they showed a reverse pattern of response to anti-TNFα treatment. Non-responders showed a progressive decrease of the percentage of CD14^+hih^CD16^+ ^monocyte subset along the study period. In contrast, responders presented a progressive increase of the percentage of CD14^+high^CD16^+ ^monocyte subset that reached healthy controls values at three months of adalimumab plus MTX treatment and was significantly higher with respect to non-responder at six months of treatment.

### The number of circulating monocytes, and of their CD14^+high^CD16^-^, CD14^+high^CD16^+ ^and CD14^+low^CD16^+ ^subsets, predicts the clinical response to anti-TNFα at three months of treatment

**Figure 3:**
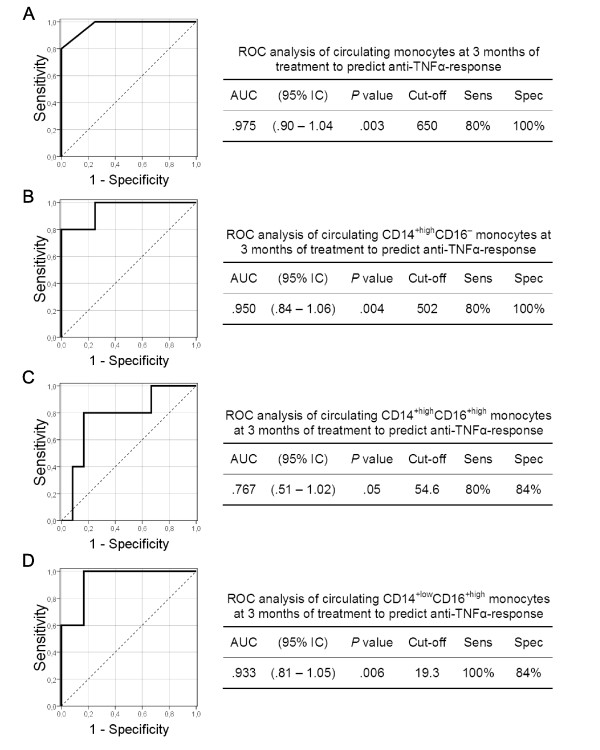
**Receiver-operating characteristic (ROC) analysis of the absolute numbers of circulating monocytes and their subsets**. Receiver-operating characteristic (ROC) analysis of the absolute numbers of circulating monocytes, and their CD14^+high^CD16^-^, CD14^+high^CD16^+ ^and CD14^+low^CD16^+ ^subsets, at three months of anti-TNFα treatment (A, B, C and D, respectively). The predictive value of the absolute numbers of monocytes was determined by calculating the area under the curve (AUC). The optimum cut-offs (cells/μl) to distinguish MTX responders from non-responders, plus their sensitivity (Sens), specificity (Spec), positive predictive value (PPV), negative predictive value (NPV) and likelihood ratio (LR), are illustrated next to the curves. These were used to verify the validation of the ROC curves and to establish the predictive power of the cut-offs.

We studied the predictive value of the absolute number of circulating monocytes, and of their CD14^+high^CD16^-^, CD14^+high^CD16^+ ^and CD14^+low^CD16^+ ^subsets, with respect to clinical response to adalimumab plus MTX. At baseline, we did not find that these biological markers show significant predictive value of the early response to anti-TNFα therapy (Figure 3). However, at three months of adalimumab plus MTX treatment, a cut-off value of 650 cells/μl for circulating monocytes was associated with 80% sensitivity, 100% specificity and a 100% PPV in terms of discriminating between eventual early responders and non-responders. A cut-off value of 502 cells/μl for the CD14^+high^CD16^- ^cell subset was associated with 80% sensitivity, 100% specificity and 100% PPV in terms of discriminating between eventual responders and non-responders. A cut-off value of 54 cells/μl for the CD14^+high^CD16^+ ^cell subset and of 19 cells/μl for the CD14^+low^CD16^+ ^subset was associated with 80% and 100% sensitivity, 84% and 84% specificity, and 100% and 83% PPV, in terms of discriminating between eventual responders and non-responders.

### CX3CR1 expression is increased in monocytes in non-responders

**Figure 4:**
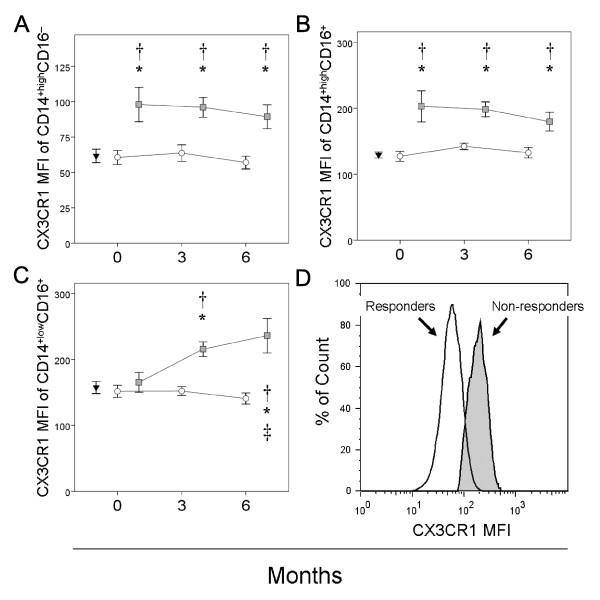
**CX3CR1 expression on circulating monocyte subsets in patients with RA at baseline and over anti-TNFα treatment**. The mean fluorescence intensity (MFI) of CX3CR1 in circulating CD14^+high^CD16^- ^(panel A), CD14^+high^CD16^+ ^(panel B) and CD14^+low^CD16^+ ^monocytes (panel C) of non-responders (□) and responders (○) at baseline and after three and six months of MTX treatment, as well as those of healthy controls (▼), are shown as means ± SEM. Panel D is a histogram of CX3CR1 for circulating CD14^+high^CD16- monocytes from a representative responder and non-responder at six months of treatment. * Significant difference between patients and healthy controls. † Significant difference between non-responders and responders. ‡ Significant difference between baseline and six-month values.

The expression of CX3CR1 was determined in the three monocyte subsets in both patients and healthy controls (Figure 4). At baseline, the expression of CX3CR1 in the CD14^+low^CD16^+ ^monocyte subset from responder and non-responders was similar and showed no significant differences with respect to healthy controls. A progressive and significant increase in CX3CR1 expression was observed in this monocyte subset in non-responders after three and six months of treatment. In contrast, the expression of CX3CR1 in CD14^+high^CD16^- ^and CD14^+high^CD16^+ ^monocyte subsets was significantly increased in non-responder patients at baseline and over the study period. No significant differences in the CX3CR1 expression were seen among CD14^+high^CD16^-^, CD14^+high^CD16^+ ^and CD14^+low^CD16^+^monocytes from responders and healthy controls.

### Similar numbers and pattern of distribution of monocytes and their CD14^+high^CD16^-^, CD14^+high^CD16^+ ^and CD14^+low^CD16^+ ^subsets in naive and MTX active patients with RA

We investigated the absolute number of circulating monocytes, and those of the CD14^+high^CD16^-^, CD14^+high^CD16^+ ^and CD14^+low^CD16^+ ^subsets as well as their frequencies of 13 active naive patients with RA, 35 MTX active RA patients and 15 healthy controls. There were no significant differences in the absolute number and distribution of circulating monocytes and their subsets between naive and MTX active patients with RA (Additional files 1 and 2).

## Discussion

This work shows that the absolute number of circulating monocytes, and of their CD14^+high^CD16^-^, CD14^+high^CD16^+ ^and CD14^+low^CD16^+ ^subsets at three months of adalimumab plus MTX treatment, have a predictive value (with high specificity and sensitivity) in terms of the clinical response after six months of anti-TNFα treatment in patients with RA. Furthermore, the pattern of abnormal redistribution of circulating monocyte subsets is similar in naive and MTX-treated active RA patients.

The treatment of RA patients with anti-TNFα biological drugs has dramatically improved the prognosis of these patients. However, a third of the treated patients do not respond to this therapy [14,15,21]. Thus, the search for biomarkers of clinical response to these agents is currently highly active. Most studies have explored the utility of genetics and autoantibody profiling to predict response to anti-TNFα therapies in RA without satisfactory results for potential clinical use [16]. A number of other potential therapeutic response biomarkers have been studied. Down-regulation of expression of a number of proinflammatory genes, including IL-1b, IL-8 and TNFAIP3, in peripheral blood mononuclear cells 72 h after the first dose of etanercept was associated with low sensitivity and specificity to a good clinical response during the first three months of treatment [22]. In addition, pretherapy CD11c gene expression on monocytes was increased in responder patients to adalimumab alone [23]. At the synovium level, the inflammatory infiltration with organized lymphocyte aggregate pattern, which may include germinal centers, has been associated with a better response to infliximab at 16 weeks [24]. Other authors have suggested that characterization of the pattern autoantibodies, serum or PBMCs' expression of cytokine levels show differences between responders and nonresponders to etanercept treatment in patients with RA [25,26]. However, at present, no robust biomarker for anti-TNFα treatment has been identified for the routine in clinical practice in RA patients. The present study, therefore, investigated the potential value of circulating monocytes, a readily accessible blood compartment, for predicting the clinical response of patients with RA to adalimumab plus MTX. The selection of these cells is based on the critical role that monocytes and tissular monocyte-driven cells plays in the induction of damage at inflamed joints and other tissues lesions [12]. The inflammatory role of circulating monocytes has been related to their ability to migrate to inflamed tissues, to provide effector functions such as cytokine and chemokine production, to undertake phagocytosis and oxidative radical generation, and to their ability to differentiate into different effector cells such as osteoclasts and dendritic cells [6,7]. Our data show that the absolute number of circulating monocytes, and of their CD14^+high^CD16^-^, CD14^+high^CD16^+ ^and CD14^+low^CD16^+ ^subsets after three months of treatment with adalimumab plus MTX, have a highly predictive value of the clinical response after six months of treatment identifying those patients with an early treatment resistance to this anti-TNFαagent.

The explanation of this observed relevance of circulating monocytes as biomarkers of adalimumab response in RA patients has been not established. However, our data support that the absolute number of the monocyte subsets play a role in the activity of the disease and in the response to adalimumab plus MTX. Our data show that the number and distribution of the monocyte subsets in naive and MTX treated active RA patients is similar. However, the treatment with MTX plus adalimumab discriminates two patterns of behavior of the monocytic compartment in RA patients. In adalimumab responders, the number of the circulating monocyte subsets normalize at three months of treatment and remain similar along the study period. In contrast, in non-responders the significant increase in the pre-treatment number of monocytes and of their subsets remains the same or even increases along the treatment. This heterogeneity in the behavior monocyte compartment cannot be ascribed to different activity of the disease because it was similar in both responders and nonresponders at the beginning of the treatment. Thus, adalimumab plus MTX treatment in responder patients is able to show an immunomodulatory effect with a drastic reduction in the number of the three monocyte subsets. Interestingly, in experimental models of RA, the depletion of circulating monocytes or synovial macrophages is associated with control of the joint inflammation and disease [27,28]. In addition, it has been reported that a third of the patients with RA with synovial effusions displays a peripheral blood monocytosis [29].

The mechanisms of the therapeutic effects of anti-TNFα on RA have not been fully established [30]. It has been shown that anti-TNFα treatment induces effects at different levels of the immunoinflammatory response, including cell trafficking reducing chemotaxis and/or leukocyte adhesion to the inflamed endothelium [31,32] and modulation of soluble mediators [33]. Interestingly, anti-TNFα agents induce apoptosis of circulating and synovial fluid monocytes in RA patients [34,35]. In addition, in RA responder patients, gene expression profiling analysis of circulating monocytes shows a decrease of chemoattractants and adhesions molecules associated with anti-TNFα treatment [36]. Furthermore, the increased number of synovial sublining macrophages is a biomarker of anti-TNFα treatment in RA [37,38]. In agreement, our data clearly support that adalimumab plus MTX treatment induces a marked decrease in circulating monocytes in responder RA patients. Moreover, we also found an increased CX3CR1 expression of circulating monocytes in non-responders. This chemokine receptor is involved in the regulation of the monocyte tissue migration. Several mechanisms might be involved in the induction of this effect of adalimumab plus MTX on circulating monocytes, including modulation of cell trafficking, monocyte survival and/or systemic generation from bone marrow precursors. The cause of the lack of effect of adalimumab in circulating monocytes of nonresponders remains unknown. The described dose dependence of the proapoptotic effect of anti-TNFα on monocytes [30] might suggest a potentially insufficient administration of adalimumab in non-responders, although a non-discovered mechanism of resistance can be also involved. These contrasting observations on apoptosis-related genes illustrated the complexity of the regulation of this process in inflammatory disease. Moreover, clinical responders to infliximab or etanercept had a greater increase in synovial apoptosis than did clinical non-responders [34,39].

The observed predictive value at three months of the absolute number of circulating monocytes, and of their CD14^+high^CD16^-^, CD14^+high^CD16^+ ^and CD14^+low^CD16^+ ^subsets, in terms of clinical response to adalimumab plus MTX treatment in patients with RA requires confirmation in large multicenter studies including patients belonging to different races. The analysis of this biomarker with other anti-TNFα agents is also required. However, the number of monocytes, and of their CD14^+high^CD16^-^, CD14^+high^CD16^+ ^and CD14^+low^CD16^+ ^subsets, in peripheral blood would appear to be a practical biomarker for predicting the response to adalimumab plus MTX in patients with RA.

## Conclusions

In summary, this study shows that the absolute number of circulating monocytes, and of their CD14^+high^CD16^-^, CD14^+high^CD16^+ ^and CD14^+low^CD16^+ ^subsets at three months of anti-TNFα treatment, have a predictive value in terms of the clinical response after six months of anti-TNFα treatment in patients with RA. Indeed, non-responder patients with RA show an increased number of monocytes and of their CD14^+high^CD16^-^, CD14^+high^CD16^+ ^and CD14^+low^CD16^+ ^subsets after three months of anti-TNFα treatment. In addition, non-responders show progressive redistribution of the monocyte subsets as well as an increased expression of CX3CR1. Thus, the number of monocytes, and of their CD14^+high^CD16^-^, CD14^+high^CD16^+ ^and CD14^+low^CD16^+ ^subsets, in peripheral blood would appear to be a practical biomarker for predicting the response to anti-TNFα in patients with RA.

## Abbreviations

7-AAD: 7-aminoactinomycin D; AUC: areas under the curves; DAS28: Disease Activity Score 28; DMARDS: disease-modifying antirheumatic drugs; EULAR: European League Against Rheumatism; FITC: fluorescein; HAQ: health assessment questionnaire; HUPA: Hospital Universitario Príncipe de Asturias; IgG: immunoglobulin G; IL: interleukin; LPS: lipopolysaccharides; LR: likelihood ratio; MoAbs: monoclonal antibodies; MTX: methotrexate; NPV: negative predictive value; PBMC: peripheral blood mononuclear cells; PE: phycoerythrin; PerCP: peridinin chlorophyll protein conjugate; PPV: positive predictive value; RA: rheumatoid arthritis; ROC: receiver operating characteristic; RPMI: Roswell Park Memorial Institute médium; SD: standard deviation; SEM: standard error of the mean; TNFα: tumor necrosis factor alpha

## Competing interests

The authors declare that they have no competing interests.

## Authors' contributions

MA-M, IS, AH and AP were responsible for the study conception and design. JC, MAS and JM were responsible for acquisition of data. AP, EC, FA and AT were responsible for analysis and interpretation of data. DD, LC and AS-A drafted the manuscript. DD and MA-M critically revised the manuscript for important intellectual content. LC and AS-A contributed to this study equally. All authors read and approved the final manuscript.

## Supplementary Material

Additional file 1 **Absolute number of circulating monocytes and of their subsets in untreated naive and MTX-treated active patients with RA and healthy controls**. The absolute number (cells/μl) of circulating monocytes (panel A), CD14^+high^CD16^- ^(panel B), CD14^+high^CD16^+ ^(panel C) and CD14^+low^CD16^+ ^monocytes (panel D) of untreated naive and MTX-treated active patients with RA and of healthy controls, are shown as the mean ± SEM. * Significant difference between patients with RA and healthy controls. Click here for file

Additional file 2 **Absolute number of circulating monocytes and of their subsets in untreated naive and MTX-treated active patients with RA and healthy controls**. Panel A represents flow cytometry analysis of circulating monocytes from a representative patient. The percentage of circulating monocytes, CD14^+high^CD16^- ^(panel B), CD14^+high^CD16^+ ^(panel C) and CD14^+low^CD16^+ ^monocytes (panel D) of untreated naive and MTX-treated active patients with RA and of healthy controls, are shown as the mean ± SEM. * Significant difference between patients with RA and healthy controls. Click here for file
